# Reliability of artificial intelligence in predicting total knee arthroplasty component sizes: a systematic review

**DOI:** 10.1007/s00590-023-03784-8

**Published:** 2023-11-27

**Authors:** Loay A. Salman, Harman Khatkar, Abdallah Al-Ani, Osama Z. Alzobi, Abedallah Abudalou, Ashraf T. Hatnouly, Ghalib Ahmed, Shamsi Hameed, Mohamed AlAteeq Aldosari

**Affiliations:** 1 Department of Orthopaedic Surgery, Surgical Specialty Center, Hamad General Hospital, Hamad Medical Corporation, PO Box 3050, Doha, Qatar; 2 Royal London Hospital, Whitechapel, London, UK; 3 Office of Scientific Affairs and Research, King Hussein Cancer Center, Amman, Jordan

**Keywords:** Artificial intelligence, Machine learning, Orthopaedics, Knee, Arthroplasty

## Abstract

**Purpose:**

This systematic review aimed to investigate the reliability of AI predictive models of intraoperative implant sizing in total knee arthroplasty (TKA).

**Methods:**

Four databases were searched from inception till July 2023 for original studies that studied the reliability of AI prediction in TKA. The primary outcome was the accuracy ± 1 size. This review was conducted per PRISMA guidelines, and the risk of bias was assessed using the MINORS criteria.

**Results:**

A total of four observational studies comprised of at least 34,547 patients were included in this review. A mean MINORS score of 11 out of 16 was assigned to the review. All included studies were published between 2021 and 2022, with a total of nine different AI algorithms reported. Among these AI models, the accuracy of TKA femoral component sizing prediction ranged from 88.3 to 99.7% within a deviation of one size, while tibial component sizing exhibited an accuracy ranging from 90 to 99.9% ± 1 size.

**Conclusion:**

This study demonstrated the potential of AI as a valuable complement for planning TKA, exhibiting a satisfactory level of reliability in predicting TKA implant sizes. This predictive accuracy is comparable to that of the manual and digital templating techniques currently documented in the literature. However, future research is imperative to assess the impact of AI on patient care and cost-effectiveness.

**Level of evidence III:**

PROSPERO registration number: CRD42023446868.

## Introduction

In this era of rapid technological evolution, artificial intelligence (AI) and machine learning (ML) have surfaced as game changers in diverse medical domains, including orthopaedic surgery [1, 2]. These advancements are notably apparent in total knee arthroplasty (TKA), an effective orthopaedic procedure that enhances patient quality of life and functional recovery [2]. TKA is one of the most frequently performed orthopaedic surgeries globally, with a projected annual surge of 85%, reaching 1.26 million by 2030 [3].

AI has found numerous applications within the domain of TKA, spanning from preoperative planning to postoperative care and monitoring [3]. In preoperative assessment, AI has been proven to offer several significant advantages. One study [4] highlighted AI's capabilities in accurately predicting parameters such as length of stay, inpatient charges, and discharge disposition [4]. Moreover, Schwartz et al. [5] demonstrated AI’s success in image recognition and classifying knee osteoarthritis using preoperative radiographs as accurately as a fellowship-trained arthroplasty surgeon [5]. The AI’s strength extends to accurately predicting component sizing, alignment, and tibial component slope with an impressive accuracy of up to 95%, a notable increase from the 72% accuracy seen with conventional methods [6, 7]. Considering the variations in patient anatomy and the wide variety of available implant designs and sizes, this precision becomes particularly valuable [6, 7]. Further demonstrating the utility of AI in preoperative planning for TKA, several studies [8, 9] revealed its capacity to employ patient demographic data, such as sex, height, weight, age, and ethnicity, to predict implant size with greater accuracy compared to radiographic templating. These findings accentuate the role of AI as an integral component in preoperative assessment and optimization for TKA. Additionally, Verstraete et al. [10] harnessed machine learning models to optimize balance and alignment during surgery. These models utilized intraoperative data to influence surgical decisions.

In postoperative care following TKA, AI’s utility is significant. For instance, Chiang et al. [11] leveraged movement monitoring sensors to continuously track patients’ range of motion progress after the surgery. This persistent tracking enables early detection of potential issues and timely interventions, optimizing the recovery and rehabilitation process. Furthermore, the image-based machine learning model showed exceptional precision in predicting postoperative complications, mirroring clinical radiographic features. A study by Lau et al. [12] highlighted that machine learning models could predict postoperative loosening of knee arthroplasty after TKA with an astounding accuracy of up to 95%.

Several recent studies have reflected the growing interest in the application of AI-based tools in knee arthroplasty and the broader orthopaedic field [2, 9, 13, 14]. Despite this, the literature reveals limitations and gaps. Most studies are single-centre explorations with a potentially biased, demographically specific focus. Compounding this, varied AI methodologies and possible overfitting of models hamper reliable comparisons and generalizations.

This systematic review aimed to synthesize the best available evidence, identify research gaps, and facilitate the effective integration of AI into TKA procedures. We hypothesize that AI can enhance the quality of care across all stages of TKA, from preoperative planning to postoperative recovery, by improving prediction accuracy and personalizing patient care.

## Methodology

This systematic review was conducted with adherence to the Preferred Reporting Items for Systematic Reviews and Meta-analyses (PRISMA) guidelines [15]. The protocol was pre-registered on the International Prospective Register of Systematic Reviews (PROSPERO); registration number: CRD42023446868.

### Search strategy

Four online databases (Ovid MEDLINE, Embase, Web of Science, and Cochrane Library databases) were searched from inception to 1 July 2023 to identify all the studies that investigated the reliability of AI in predicting accurate intraoperative TKA component sizes. The following keywords were included: Artificial Intelligence OR Machine learning AND Orthopaedics AND Total knee arthroplasty.

### Eligibility criteria

Studies were considered eligible if they satisfied the following criteria: (1) reporting accuracy or reliability of AI in predicting TKA components sizes, (2) all types of TKA (regardless of the design or manufacturer), (3) all types of AI models (machine or deep learning), and (4) published in the English language.

Exclusion criteria included (1) failure to report accuracy specifically related to AI in TKA, (2) correlating AI tools with other conditions than TKA, (3) studies with incomplete or unextractable data for review, and (4) review articles, preclinical, and case reports.

### Study screening

Two authors conducted the screening process independently and blindly by screening the titles and abstracts of the retrieved articles. For studies meeting the pre-specified eligibility criteria, full-text review was performed. Any disagreement between the two authors was resolved by discussion with a more senior author.

### Data abstraction

Two authors independently extracted the data from included articles. The following data were collected: studies’ characteristics, patients’ demographics (such as age, sex, and body mass index), implant designs, manufacturer, AI model type (machine or deep learning) and used algorithms, type of AI validation, accuracy measurements in the form of mean absolute error (MAE), root-mean-square error (RMSE), coefficient of determination (R2), exact size, + -1, + -2 sizes, and overall accuracy. The primary outcome was + -1 size accuracy. Secondary outcomes were other measures of accuracy.

### Quality assessment

Two authors conducted the methodological quality assessment blindly and independently using the Methodological Index for Non-Randomized Studies (MINORS) criteria [16]. According to the MINORS criteria, comparative and non-comparative studies can achieve a maximum score of 24 and 16, respectively. Comparative studies are graded as very low quality (0–6), low quality (7–10), fair quality (11–16), good quality (16–20), and high quality (> = 20). Noncomparative studies are grade as very low quality (0–4), low quality (5–7), fair quality (8–12), and high quality (> = 13) [16]. Table 1 demonstrates the quality assessment of the included studies using MINORS criteria.

**Table 1:** MINORS criteria of the included studies

Study/items	A clearly stated aim	Inclusion of consecutive patients	Prospective collection of data	Endpoints appropriate to the aim of the study	Unbiased assessment of the study endpoints	Follow-up period appropriate to the aim of the study	Loss to follow-up less than 5%	Prospective calculation of the study size	Total
Kunze [9]	1	1	2	2	0	2	2	0	11
Kunze [13]	2	2	1	2	1	2	2	0	12
Lambrechts [2]	2	2	1	2	1	2	2	0	12
Burge [14]	2	1	1	2	0	2	2	0	10

## Results

### Evidence synthesis/study selection

**Fig. 1:**
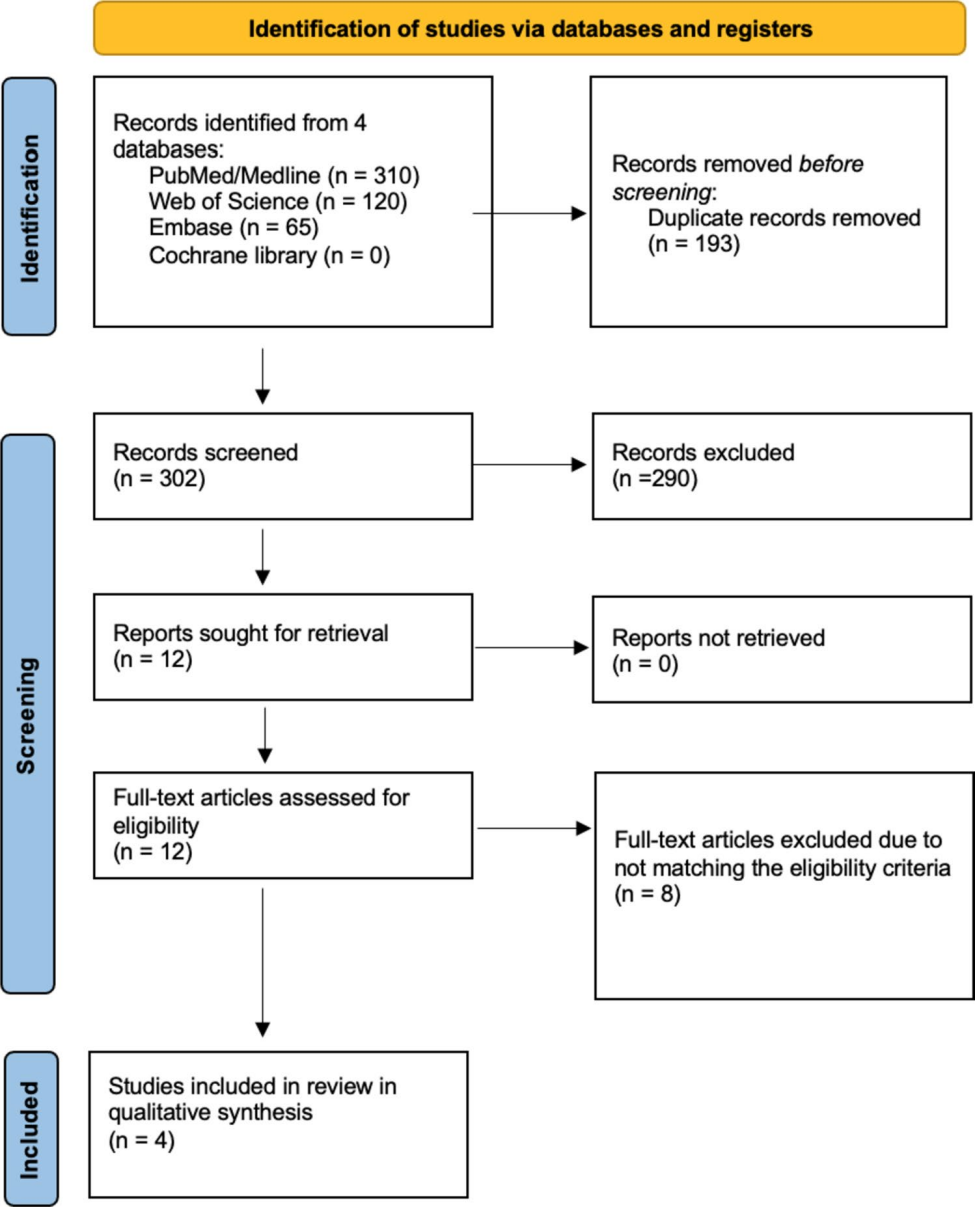
PRISMA flow diagram of record identification, screening, and selection in systematic review and meta-analysis

A primary search of databases yielded a total of 495 articles. After removing duplicates, a total of 302 were subjected primary screening by title and abstract. Of the screened articles, 12 papers were deemed relevant and subjected to a secondary screening process (i.e. full-text evaluation). The result of such a process left four articles in the final qualitative synthesis. The PRISMA flowchart is displayed as Fig. 1.

### Characteristics of included studies

A total of four articles comprised of at least 34,547 patients were included in this review. All included studies were published between 2021 and 2022. The studies primarily originated from the USA, the UK, and Belgium. Of the included articles, only two reported using the TRIPOD guidelines for AI model development and validation. Nine various AI-based algorithms were used. A total of four models were utilized to preoperatively predict femoral and tibial TKA implant sizes. On the other hand, one model was developed to predict number of corrections made to patient-specific or surgeon preoperative plans. Baseline characteristics of included studies are presented in Table 2.

**Table 2:** A summary of baseline study characteristics

Study	Design, LoE	Country	No. of patients	Implant type	Manufacturer (Zimmer, Stryker, DePuy..)	Age (Y)	Height (cm)	Weight (Kg)
Kunze [13]	Case control, III	USA	17,283	PS, CR	Biomet, Corentic, Exactech, DePuy, Stryker, Microport, and Zimmer	66.3 ± 9.4	169.3 ± 10.8	91.3 ± 21.0
Kunze [9]	Case control, III	USA	11,777	NR	Stryker	66.5 ± 9.5 (17–94)	169.6 ± 10.8 (116.8–210.8)	90.0 ± 19.4 (30.8–181.4)
Lambrechts[2]	Case control, III	Belgium	5409	NR	Vanguard, Personna, and Zimmer	NR	NR	NR
Burge [14]	Case control, III	UK	78	NR	Zimmer, DePuy, Smith and Nephew, Maxx Orthopaedics, and Stryker	46 -79	NR	NR

Study	BMI (kg/m^2^)	Gender (M:F)	AI model used	No. of algorithms (with names)	Type of validation	Index test	Reference standard	Data source	Conclusion
Kunze [13]	31.9 ± 6.4	7421:9862	Machine learning	5 (stochastic gradient boosting, random forest, support vector machine, extreme gradient boosting, and elastic-net penalized logistic regression)	Training set 80% Test Set 20%	XR	Actual sizing from OR records	Two large tertiary academic and six community hospitals	Novel machine learning algorithms demonstrated good to excellent performance for predicting TKA component size. Patient sex appears to contribute an important role in predicting TKA size
Kunze [9]	31.2 ± 5.6 (13.7–59.8)	5306:6472	Machine learning	5 (stochastic gradient boosting, random forest, support vector machine, extreme gradient boosting, and elastic-net penalized logistic regression)	Training set 80% Test set 20%	XR	Actual sizing from OR records	Two large academic and three community centres	Machine learning algorithms demonstrated good accuracy for predicting within one size of the final tibial and femoral components used for TKA. Patient height and sex were the most important factors for prediction femoral and tibial component size, respectively
Lambrechts[2]	NR	NR	Machine learning	3 (multi-task LASSO (MTL), LASSO, and group LASSO)	Training set 70% Test set 30%	MRI	Actual sizing from OR records	39 experienced surgeons from 38 hospitals	A machine learning-based preoperative plan, which captures surgical preferences in a patient- and surgeon-specific manner, has the potential to reduce the time needed to modify the preoperative plan prior to approval
Burge [14]	NR	33:45	Machine learning	1 (ML-based 2D–3D pipeline)	Training set 90% Validation set 10% Test set (additional subjects 78) 44%	XR & MRI	Actual sizing from OR records	Osteoarthritis Initiative (OAI) and KISTI	Higher prediction accuracies than generally reported for manual templating techniques

*LoE* Level of evidence, *BMI* Body mass index, *ML* Machine learning, and (*Y*) Years

### Prediction of femoral TKA implants

Kunze et al. [9] investigated the effectiveness of five different machine learning (ML) algorithms in the prediction of TKA implants. The models were trained on 13,828 patients and tested on 3455 patients. The five ML models included random forest (RF), support vector machine (SVM), stochastic gradient boosting (SGB), elastic-net penalized linear regression (ENPLR), and extreme gradient boosting (XGB). The SGB model was the best performing model for femoral component size prediction with a mean absolute error and root-mean-squared error (RMSE) values of 2.32 and 2.94, respectively. The accuracy of model for the implant’s ± 4 mm size was 83.2%. Moreover, the exact bucket, within ± 1 bucket, and within ± 2 bucket sizes accuracy of the model were as follows: 48.2%, 95.0%, and 99.8%.

Kunze et al. [13] also explored the performance of five different ML models in TKA implant prediction. The models were tested on 11,777 patients. The authors found that the SVM model yielded the best performance parameters for femoral implant prediction with a mean absolute error and RMSE of 0.73 and 1.06, respectively. The model’s accuracies at exact size, within ± 1 size, and within ± 2 sizes of the actual implant size were 42.2%, 88.3%, and 97.6%. Height was deemed the variable with the highest relative influence on component size.

Burge et al. [14] tested the performance utility of a ML-based 2D–3D pipeline which is able to generate accurate predictors of distal femur and proximal tibia bones from X-ray images. The model was trained on data provided from the Osteoarthritis Initiative (OAI) and Korea Institute of Science and Technology Information (KISTI) databases. The tool was tested on 78 patients and five different generic TKA components including Zimmer Biomet (NexGen), DePuy (Sigma), Smith & Nephew (Legion), Maxx Orthopaedics (Freedom), and Stryker (Scorpio). The authors demonstrated that the mean RMSE percent prediction for femoral component size was 77.9%. The accuracy reaches 99.7% for within ± 1 size metrics. Moreover, the tool was able to predict 71.8% of maximum over/under hang; this accuracy increased to 99.5% at a ± 1 size metric. A summary of the accuracy measurements across various included AI models is displayed in Table 3.

**Table 3:** Summary of various included AI models accuracy measurements in predicting TKA component sizes

	Accuracy (femoral component)						Accuracy (tibial component)					
Study	MAE	RMSE	R2	Exact size	± 1 size	± 2 size	MAE	RMSE	R2	Exact size	± 1 size	± 2 size
Kunze [9]	A:2.32/B: 2.30./C: 2.32/D: 2.32/E: 2.32	A: 2.94/B: 2.96/C: 2.95/D: 2.94/E: 2.95	A: .61/(B–E):.60	A: 48.2%/B: 49%/C: 48.6%/D: 48.4%/E: 48.4%	A: 95%/B: 94.3%/C: 94.8%/D: 95%/E: 94.8%	A: 99.8%/B:99.7%/C:99.7%/D: 99.8%/E: 99.8%	A:2.35/B: 2.34/C: 2.31/D: 2.34/E: 2.35	A: 3.04/B: 3.06/C: 3.05/D: 3.04/E: 3.05	A: .68/B: .67/C–E: .68	A: 58.4%/B: 59.5%/C: 60%/D: 58.7%/E: 58.9%	A: 97.8%/B: 97.3%/C: 97.9%/D: 97.7%/E: 97.7%	A: 99.9%/B:99.8%/C:99.9%/D: 99.9%/E: 99.9%
Kunze [13]	A:0.74/B: 0.73/C: 0.73/D: 0.74/E: 0.74	A: 1.06/B: 1.07/C: 1.06/D: 1.06/E: 1.06	A: 0.57/B: 0.55/C: 0.56/D: 0.57/E: 0.56	A: 41.1%/B: 42.1%/C: 42.2%/D: 40.9%/E: 41.6%	A: 88.2%/B: 88.2%/C: 88.3%/D: 88.1%/E: 88.2%	A: 97.6%/B: 97.5%/C:97.6%/D: 97.7%/E: 97.5%	A: 0.70/B: 0.71/C: 0.70/D: 0.69/E: 0.70	A: 1.04/B: 1.06/C: 1.04/D: 1.04/E: 1.03	A: 0.63/B: 0.62/(C–E): 0.63	A: 43.7%/B: 43.8%/C: 44.2%/D: 44.5%/E: 43.8%	A: 89.5%/B: 88.9%/C: 89.7%/D: 89.6%/E: 90%	A: 97.6%/B: 97.5%/C:97.7%/D: 97.6%/E: 97.7%
Lambrechts [2]	NR	NR	NR	82.20%	NR	NR	NR	NR	NR	85.00%	NR	NR
Burge [14]	NR	Mean 77.95%	NR	77.95%	99.74%	NR	NR	80.51%	NR	80.51%	99.74%	NR

*A* Stochastic gradient boosting, *B* Random forest, *C* Support vector machine, *D* Extreme gradient boosting, and *E* Elastic-net penalized logistic regression. *MAE* Mean absolute error, *RMSE* Root-mean-square error, and *R2* Coefficient of determination

### Prediction of tibial TKA implants

Kunze et al. [9] demonstrated that the SBG ML model had the best performance in predicting tibial component size. The model’s mean absolute error and RMSE are as follows: 2.35 and 3.04, respectively. The accuracy of model for the implant’s ± 4-mm size was 83.0%. At the exact bucket, within ± 1 bucket, and within ± 2 bucket sizes, the model exhibited the following accuracies: 58.4%, 97.8%, and 99.9%. The SGB predictions of patient’s tibial component size were primarily influenced by patients’ biological sex.

Kunze et al. [13] showcased that the ENPLR ML model had the best performance in predicting tibial component size. The model yielded a mean absolute error and RMSE values of 0.70 and 1.03, respectively. The model’s exact size accuracy was 43.8%, which increased to 90.0% and 97.7% at the within ± 1 size and within ± 2 sizes thresholds, respectively. Biological sex was the most influential factor in affecting the ENPLR model predictions of tibial size.

Burge et al. [14] examination of an ML-based 2D–3D pipeline tool revealed that their model is able to predict tibial sizes with an 80.5% accuracy in terms of RMSE and 71.8% in terms of maximum over/under hang. The RMSE and maximum over/under hang accuracy increase to 99.7% and 99.9% within a ± 1 size threshold, respectively. Patients’ age did not correlate with the model’s prediction accuracy (Table 3).

### Prediction of patient-specific preoperative planning corrections

Lambrechts et al. [2] tested the performance of ML models in the prediction of preoperative planning corrections for the femur–tibia joint interface. The ML models were validated on a dataset comprised of 5409 patients undergoing TKA. A 70:30% ratio was utilized for training/cross validation and testing, respectively. ML-based models included multi-task LASSO (MTL), LASSO, and group LASSO. All three models were supplemented with either support vector regression (SVR) or least absolute deviation support vector machines (LAD–SVR) for conducting regression analysis. The authors found that, only average, AI-based preoperative plans resulted in a 39.7% improvement compared to manufacturer preoperative plans. Improvement was measured as the percentage reduction in correction in the AI-based model compared to that of the manufacturer (3.76 vs. 7.13). The best ML model was the combination of LASSO with LAD–SVR among most cases. Nonetheless, all included models resulted in significant improvements compared to the manufacturers’ plans. Interestingly, there was a moderate positive correlation between number of corrections made by surgeons on the manufacturer’s plan and performance improvement using ML. Overall, compared to manufacturer plans, AI-based plans resulted in significantly higher implant accuracy of 82.2% and 85% for both femoral and tibial implant sizes, respectively.

## Discussion

The main findings of this review were that the accuracy of predicting TKA femoral component sizing spanned from 88.3 to 99.7% within a one size deviation, while the accuracy for tibial component sizing ranged from 90 to 99.9% ± 1 size. As Kunze et al. have demonstrated, predictive AI modelling has the ability to more accurately predict component sizing, in comparison with pre-existing statistical modelling from manufacturers [9]. The advantage of AI in this context is the ability to incorporate multiple discrete, ordinal, and continuous variables into the predictive model, with underlying machine learning refining the model contemporaneously. This provides an opportunity for real-time model refinement, and thus, AI can accommodate both for changing surgical, implant, and patient factors [17]. Within this review, predictive AI model accuracy in estimating component sizing has been demonstrated across substantial datasets; a finding which merits further research focuses from the wider orthopaedic community [2, 9, 14].

Utilization of predictive AI-driven modelling in TKA can be seen as an opportunity to economize procedure-related costs. Within TKA surgery, AI modelling in the preoperative context could enable TKA surgery to be delivered in resource-constrained environments. By processing demographic and radiographic data across a single population, there remains the inherent possibility of streamlining surgical implant kits and thus reducing commercial cost to health-care providers [18]. Without the need for an extensive implant inventory, TKR surgery could be considered even more accessible than present, with a reduction in implant costs allowing for more equitable access to elective orthopaedic care [19, 20].

With the inherent possibility of utilizing AI in TKA to streamline implant inventory, tangible environmental benefits could also be realized [18, 21]. Currently, the use of TKA kits with an extended implant inventory present both increased manufacturing and sterilization costs, both for implant makers and consumers [18, 21]. Preoperative AI modelling executed with a high degree of accuracy could support the production of demographic-specific implant kits and thus keep the need for extensive surgical inventory in TKR for either complex deformity or revision operations.

The use of substantial datasets in all included studies enhances the credibility of study conclusions. By utilizing large datasets, the AI modelling was able to understand both a wider demographic than traditional manufacturer information and incorporate more clinically relevant demographic data into the existing AI structure [22]. Given the advent and utility of registry data in global orthopaedics, utilizing existing registry frameworks to enable AI-related research should allow for rapid research into this field [23]. The ability of AI to harness ‘big data’ through pre-existing joint registries, both retrospectively and prospectively, should help to prove AI’s utility in this sphere, and provide further demographic data to solidify existing models [24, 25].

Fundamentally, variability in alignment and operative principles from within the orthopaedic community presents a unique challenge when developing an all-encompassing AI model in TKR surgery [2, 26]. The variability in operative principles employed by each surgeon may add uncertainty to any AI model, as the reasoning for intraoperative decisions that are fed back into the model is inconsistent between individual surgeons [2, 26]. A possible future endeavour would focus on developing models for distinct groups of TKA surgeons, for examples those practicing with a specific implant, alongside, for instance, utilizing mechanical alignment principles. In this context, repeatable and consistent intraoperative decision-making could be incorporated into existing AI infrastructure.

Future work should focus on well-designed randomized studies that compare traditional techniques to AI preoperative planning, with a focus on both eventual implant choice and ultimately subsequent patient outcomes. Currently, AI applicability in TKA can be seen as a possible cost-saving solution; however, the tangibility of clinical benefit remains unclear within the published literature. AI in TKA surgery should be outcomes-focused, with consideration given both to patient-reported outcome measures and radiological outcomes [27]. Further, current AI models presented within this review lack rigorous external validation, and this should be sought prior to widespread clinical use.

Within the included literature, references are made to a theoretical intraoperative time saving using AI prediction; however, these data have not been captured within these studies. Further, AI in TKA has been employed primarily in predicting implant sizing in primary TKA. Extrapolation of these results to patients with severe deformity or requiring revision TKA patients warrants caution, as these patient cohorts present unique perioperative surgical considerations [28].

### Limitations of the current literature

The applicability of AI-based models in TKA is still in its infancy due to the following limitations: First, lack of external validation of proposed models. Second, failure to account for other factors affecting TKA implants such as tibial slope, degree of constraint, revision components, etc. Third, AI-based model should be validated for a number of commonly used manufacturers, which may not be the case for most studies. Fourth, the sampling strategies of included studies may not represent all TKA patients; thus, future studies should attempt to account for patient heterogeneity. Fifth, most studies lack a control group/intervention by which the results of AI-based predictions are compared against. Sixth, there is significant variability in terms of model development and reporting. Therefore, black-box models may not be relevant for all TKA populations. Finally, inter- and intra-surgeon variability was not account for in any of the proposed models.

## Conclusion

This study highlights AI’s potential as a valuable adjunct to TKA planning, demonstrating a reliable ability to predict implant sizes comparable to the accuracy of manual templating methods found in the current literature. However, it remains crucial for future research to evaluate AI’s impact on patient care and cost-effectiveness.

## Data Availability

Available upon request.
